# Reconstruction of Coracoclavicular Ligaments with Semitendinosus Autograft and Temporary Kirschner Wires is a good option for Chronic Acromioclavicular Joint Instability

**DOI:** 10.5704/MOJ.2403.013

**Published:** 2024-03

**Authors:** A Ulusoy, N Turgut, F Cilli, AM Unal

**Affiliations:** 1 Department of Orthopaedics and Traumatology, Acibadem University, Mugla, Turkey; 2 Department of Orthopaedics and Traumatology, Baskent University, Adana, Turkey; 3 Department of Orthopaedics and Traumatology, Private Meddem Hospital, Isparta, Turkey

**Keywords:** acromioclavicular joint, acromioclavicular separation, coracoclavicular ligament reconstruction, acromioclavicular reconstruction

## Abstract

**Introduction:**

This study reports the results of surgical anatomic reconstruction of torn coracoclavicular ligaments with an autogenous semitendinosus graft and temporary Kirschner wires (K-wires) in chronic acromioclavicular (AC) joint dislocations.

**Materials and methods:**

Nineteen shoulders underwent surgical anatomic reconstruction of torn coracoclavicular (CC) ligaments with an autogenous semitendinosus tendon graft and temporary K-wires for Rockwood grade III, IV and V chronic AC joint dislocations. Pre-operative data included patients’ demographic characteristics, injury characteristics and surgical histories. The primary outcome measures were the University of California Los Angeles (UCLA) shoulder rating scale and visual analogue pain scoring (VAS), and the complications were noted for each patient.

**Results:**

Surgical anatomic reconstruction of torn CC ligaments was performed in 19 patients with a mean age of 41.6±16 years (range 21–72 years). All of the patients were satisfied and felt better after CC ligament reconstruction. The average UCLA shoulder rating scale score was good/excellent: 29.4 (range 23–34) out of 35 points. The average pre-operative VAS score was 7.7 points out of 10 and improved to 1.1 points post-operatively (p<0.05). None of the patients experienced failure during the follow-up. One patient had a mild subluxation, but the patient was satisfied with the result.

**Conclusions:**

This technique is simple, reliable, and biologic without major complications. It is also a cost-effective procedure since it can be performed with Kirschner wires and autogenous grafts. It has a major advantage of leaving no implants inside the joint, which can lead to hardware complications, and it can be performed in basic operating room settings.

## Introduction

Acromioclavicular joint dislocation (ACD) is one of the most common shoulder injuries and is usually sports related^1,2^. Treatment for ACD is based on the widely accepted Rockwood classification system^3^. Grades I and II are conservatively treated, generally with good success. Grades IV to VI dislocations require operative treatment, while the treatment choice for grade III ACD remains a matter of debate. Chronic acromioclavicular joint-related symptoms, such as pain, weakness, discomfort and cosmetic dissatisfaction, can occur regardless of which method is utilised for the management of ACD^4^. Chronic symptomatic patients (>6 weeks from the initial injury) and those who have undergone failed surgery are potential candidates for surgical management.

There is no consensus regarding which surgical method is most appropriate for chronic ACD. While the Weaver–Dunn procedure is the most popular choice^5,6^, the coracoacromial ligament’s inherent weakness and unreliability in maintaining the reduction have led surgeons to use stronger grafts for reconstruction and add temporary or permanent fixation due to the high failure rates^7,8^. Consequently, anatomic coracoclavicular (CC) ligament reconstruction with autogenous tendon grafts has become an increasingly popular treatment option^9,10^. Tendon grafts, such as semitendinosus, tibialis anterior and palmaris longus grafts, have been shown to mimic native CC ligaments in biomechanical studies^11,12^. In particular, the semitendinosus tendon graft (STG) is an easily feasible, cost-effective and viable choice for ACD treatment. Temporary fixation methods, including the Kirschner wire (K-wire), cerclage wire, screw, hook-plate fixation, suture fixation, coracoid transfer and anchors, may be added to the reconstruction based on the preferences of the operating surgeon^13,14^.

This retrospective study reports the clinical and functional outcomes of anatomic CC ligament reconstruction (Rockwood grades III, IV and V) using an autogenous STG and temporary fixation with a K-wire in chronic ACD patients.

## Materials and Methods

After receiving approval from the local ethics committee, written informed consent was obtained from all participants. This study evaluated 21 patients who underwent surgery for ACD between 2005 and 2013 performed by the same surgeon (FC). Torn CC ligaments were reconstructed using STG. Of these patients, two were excluded from the study due to inadequate follow-up. The clinical, functional and radiological results of the remaining 19 patients were analysed (Table I). The inclusion criteria were as follows: (i) grade III–V ACD, (ii) CC ligament reconstruction with autogenous STG, (iii) at least six-month follow-up, (iv) age at least 18 years and (v) at least six-week-old initial trauma. Patients who had sustained an ipsilateral upper extremity injury, had a neurologic disorder or were unable to cooperate were excluded. All patients were examined with bilateral anteroposterior clavicle views and standing Zanca views both pre-operatively and post-operatively. The dislocations were classified according to the Rockwood classification system.

**Table I:** The demographic and clinical data of the patients.

Patients	Age Sex	Previous treatment	Etiology	Timing of reconstruction after initial trauma	Postop UCLA shoulder rating scale	Preop VAS Score	Score VAS Score	Complication
1	23 ♂	Acute pinning	Motor vehicle accident	6 weeks	33	8	1	Superficial pin-tract infection
2	34 ♀	Velpeau bandage	Sports injury	7 weeks	29	8	1	No
3	31 ♂	Velpeau bandage	Sports injury	6 weeks	29	9	2	No
4	59 ♂	No treatment	Simple fall	5 months	28	6	0	No
5	41 ♀	Acute pinning	Sports injury	8 weeks	34	8	0	No
6	65 ♂	Velpeau bandage	Simple fall	8 weeks	23	7	3	Mild residual pain
7	33 ♂	Acute pinning	Sports injury	8 weeks	29	8	1	Superficial pin-tract infection
8	62 ♂	Velpeau bandage	Simple fall	10 weeks	23	8	2	Mild residual pain
9	28 ♂	Acute pinning	Simple fall	8 weeks	34	9	1	No
10	44 ♀	Acute pinning	Motor vehicle accident	6 weeks	32	9	1	Asymptomatic subluxation
11	21 ♂	Velpeau bandage	Sports injury	6 weeks	29	8	1	No
12	48 ♂	Acute pinning	Simple fall	8 weeks	28	7	2	No
13	26 ♀	Velpeau bandage	Sports injury	10 weeks	32	6	1	Superficial pin-tract infection
14	37 ♂	Velpeau bandage	Sports injury	8 weeks	33	8	1	No
15	23 ♂	Acute pinning	Simple fall	8 weeks	28	7	1	No
16	66 ♂	Velpeau bandage	Simple fall	11 weeks	28	7	1	No
17	72 ♂	Velpeau bandage	Simple fall	6 weeks	28	8	0	No
18	35 ♂	Velpeau bandage	Sports injury	6 weeks	31	6	1	No
19	43 ♂	Acute pinning	Simple fall	3 months	29	8	1	No

**Fig 1:**
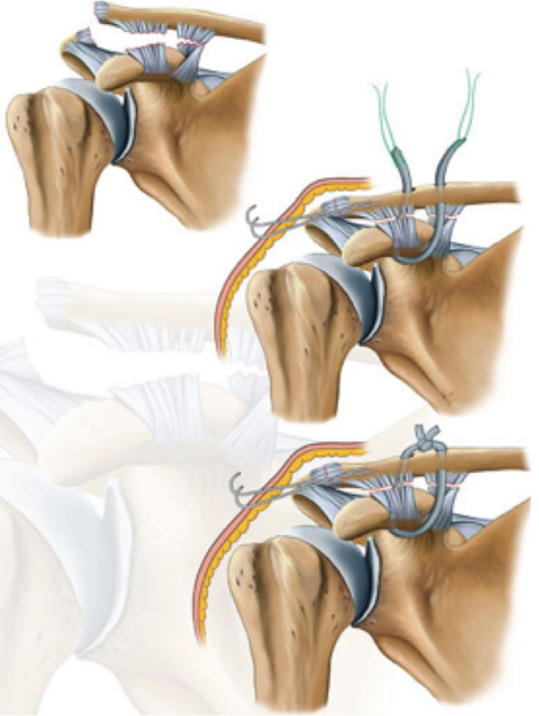
The semitendinosus graft passing, clavicular tunnel positioning and tying over the top of the clavicle to maintain reduction.

An oblique skin incision was made, extending from the distal clavicle to the coracoid, under general anaesthesia in the beach-chair position. After splitting the muscle layers, the coracoid was reached through the anterior of the clavicle by blunt dissection. Two 2.4-mm tunnels corresponding to the origins of the conoid and trapezoid ligaments were created through the distal clavicle, approximately 1.5cm apart from each other. The distal tunnel was 2.5cm away from the AC joint. After harvesting the STG from the ipsilateral leg, the graft was prepared with no. 2 Ethibond sutures [Ethibond, Somerville, NJ] and passed beneath the coracoid. Both ends of the graft were also passed inside out through the tunnels after enlarging the tunnels (Fig. 1). The AC joint was provisionally reduced with two 1.5/1.8-mm smooth K-wires while keeping the graft ends under adequate manual tension (Fig. 1). The ends of the graft were sutured onto themselves and the surrounding soft tissues (Fig. 1). The layers and the skin were closed in a routine fashion, leaving a mini-hemovac drain in place after the fluoroscopic control of AC joint stability and bleeding.

The same rehabilitation protocol was applied to all patients. A shoulder sling was used for six weeks after the operation. Active range of motion (ROM) was restricted, but passive ROM exercises were initiated as early as possible after achieving adequate pain control, considering the patient’s tolerance. K-wires were removed at the end of the sixth week. Subsequently, physical therapy sessions were commenced for active ROM of the shoulder joint and controlled muscle strength exercises. Heavy weightlifting was restricted for an additional six weeks. Patients were evaluated at the end of the third and sixth months and at the end of the first year. Following this, the patients were asked to attend annual follow-up visits if they had persistent complaints.

The primary outcome of this study was the clinical and functional evaluation, which was based on the University of California Los Angeles (UCLA) shoulder rating scale^15^. Pain status was determined by visual analogue pain scoring (VAS) (0–10). The scores were obtained from the patients’ medical records. Complications, including AC joint arthritis, surgical failure, infection and donor site morbidity, were also noted for each case.

The statistical analyses were performed with SPSS 20.0 [IBM Inc., Chicago, IL, USA]. The descriptive statistics were presented as mean ± SD and frequency (percentage) for all variables. A nonparametric test, the Wilcoxon signed ranks test, was chosen for statistical analysis, as the sample size was less than 30. In all analyses, p<0.05 value was considered a statistically significant result.

## Results

Nineteen patients (14 males, 5 females) with a mean age of 41.6 years (range, 21 to 72) were followed-up for a mean length of 14 months (range 6–33 months). The average time from initial trauma to the reconstruction was 8.5 weeks (range 6–20 weeks). Initial treatment consisted of conservative treatment with bandaging for 10 patients and acute pinning for eight patients. One patient received no initial treatment. The patients who were conservatively followed had initially been recommended for surgery but had declined the surgical option. Two patients sustained injuries due to motor vehicle accidents, eight had sports-related injuries and nine experienced injuries from simple falls.

All patients reported satisfaction and felt better after CC ligament reconstruction. The average UCLA shoulder rating scale was rated as good/excellent: 29. 4 (range 23–34) out of 35 points. The average pre-operative VAS score was 7.7 (range 6–9) out of 10 points, which significantly decreased to an average post-operative VAS score of 1.1 point (range 0–3). According to the nonparametric Wilcoxon signed ranks test, pre-operative VAS scores improved significantly (p<0.05) in terms of pain relief.

No donor site-related complications were observed. Three patients had superficial pin-tract infections, which were healed with oral antibiotics and routine wound care. One patient had a mild residual AC joint subluxation, but the patient was satisfied with the result (tenth patient in Table I).

Four patients had radiologic signs of degenerative arthritis of the AC joint pre-operatively (sixth, eight, sixteenth and eighteenth patients in Table I). Two of them (sixth and eighth patients in Table I) had mild residual pain during strenuous or specific activities, requiring conservative treatment measures, including oral pain medication, rest and occasional cold application. Their UCLA rating scale were fair/poor (23 points).

The sixteenth and seventeenth patients were 66 and 72 years old, respectively, and both had arthritic signs on plain radiographs. Given the advanced age, presence of AC joint arthritis and persistent post-operative pain of the sixth and eighth patients, additional excision of the distal clavicle were performed for these patients. The outcomes of these two patients were assessed as good/excellent, achieving a UCLA rating scale of 28 points.

## Discussion

This study shows that surgical anatomic reconstruction of torn CC ligaments with an autogenous STG and temporary fixation of the AC joint with K-wires is a reliable and satisfactory surgical option for chronic patients with a low recurrence rate. The technique described here has a major advantage of not leaving any implants inside the joint, which could result in complications due to implant or bone fatigue. It is also a simple, cost-effective, biologic and dynamic option for the treatment of chronic AC joint instability, and it can be performed in any basic operating room setting.

Numerous surgical options for the treatment of chronic AC joint dislocation (ACD) have been proposed in the literature, but no clinical guidelines or algorithms have been established for this issue. The published data lack quality, and only case series with small sample sizes have been presented. The treatment strategies can be classified based on CC ligament healing or reconstruction. Due to the inefficient healing capacity of CC ligaments in chronic situations, the literature, which includes no level I studies, favours reconstruction surgeries^6^. The most popular reconstruction technique is the Weaver–Dunn procedure, which includes transfer of the coracoacromial ligament (CAL) to the lateral end of the distal clavicle^5^. Although this surgery and its various modifications usually yield good functional results, complications and high recurrence rates are frequently observed due to CAL weakness^16^. Therefore, reconstruction with tendon grafts has been increasingly replacing the Weaver–Dunn procedure. Semitendinosus tendons have high tensile strength and result in stable grafts with abundant graft length^17^. STG has been our preference for CC reconstructions since biomechanical studies proved its suitability, and we used an autogenous STG in all ACD patients in this study. Costic *et al* found that STGs were biomechanically superior to CAL and more closely resembled the characteristics of native CC ligaments^11^. Mazzocca *et al* also compared hamstring tendons with CAL in their cadaveric study and proposed their usage to decrease recurrence, residual pain and enable early rehabilitation^7^. In their study, they demonstrated that when an anatomical coracoclavicular reconstruction is performed with a tendon graft, there is reduced anterior and posterior translation, and it closely resembles the intact state. This horizontal stability is normally obtained by the intact superior–posterior capsuloligamentous complex, which becomes non-functional in chronic ACD^18^. In this study, none of the patients experienced failure, and only one patient had mild residual subluxation. Hegazy *et al* reported a 30% failure rate in Weaver–Dunn surgeries, whereas none of the STG group experienced failure, consistent with the present study^19^. We believe that the superiority of STGs contributed to the low recurrence rate.

Some authors have suggested using temporary fixation methods during STG remodelling. These treatment methods may have disadvantages regarding hardware complications. The risk of hardware migration for K-wires, the need for implant removal due to hardware irritation and pain, acromial osteolysis and fractures at the implant site due to hook plates and CC screws are potential disadvantages^16^. Suture loops may be inserted arthroscopically, but they involve the risk of cutting through the bone^20^. The authors of the current study prefer using K-wires to ensure proper graft incorporation for a brief six-week period after surgery. The end of the Kirschner wires is left outside the skin and bent to prevent migration. In three cases, superficial pin tract infections occurred. However, they were successfully managed with simple antibiotic treatment without the need for additional surgical intervention. STGs also have the advantage of eliminating the risks of non-biologic implants, such as foreign body reactions, because they do not require any augmentation.

Successful restoration of the AC joint can be achieved by following the footprints of ligament insertions. Nearly all newer anatomic reconstructions seek to better recreate the native anatomy. There is no current consensus on the number or size of coracoid and clavicular tunnels, as there is for graft choice and fixation method. The double clavicular tunnel technique is generally preferred to improve the kinematics of the AC joint^12^. Cadaveric studies demonstrated that the trapezoid and conoid ligament insertions are 2.5cm and 4.6cm away from the lateral border of the clavicle, respectively^7,21^. In our study, clavicular bone tunnels were also positioned 1.5cm apart to mimic the native anatomy. The distal tunnel was positioned 2.5cm medially from the distal end of the clavicle. The use of bone tunnels in this technique, increase the risk of post-operative fracture and decrease the strength of the clavicle. In fact, clavicle fracture is a serious complication associated with anatomic reconstruction of the AC joint. Millet *et al* presented a review of 12 studies that reported complications following anatomic CC ligament reconstruction with biologic grafts, finding an overall complication rate of 39.8%^22^. The most serious complications are graft failures, hardware complications and distal clavicle and/or coracoid fractures because of the bone tunnels. Martetschlager *et al* reported two clavicle fractures and one coracoid fracture, which were associated with 6-mm bone tunnels^23^. The presented technique minimises the risk of clavicle fracture by using 2.4-mm bone tunnels created on the distal clavicle and by applying a looping technique around the coracoid to avoid coracoid fracture. Millet *et al* advocated for anatomic CC ligament reconstruction, utilising an allograft loop around both the coracoid base and distal clavicle. They used 3-mm bone tunnels, avoiding the use of large bone tunnels (>5mm), with the goal of reducing either clavicle or coracoid fractures^22^. The looping technique is thought to reduce the risk of coracoid fractures.

The technique we used was not associated with many complications. There were only three superficial pin-tract infections, and they were treated and healed with oral antibiotics. The only patient who had a mild residual AC joint subluxation was satisfied with the result of the treatment and did not require a further operation. None of the surgeries resulted in failure. Donor site morbidity is the main disadvantage of STG. Saphenous nerve injury, decreased hip adductor strength, decreased knee flexion and internal rotation strength and hypotrophy of thigh muscles are the possible adverse effects of STG harvesting^24^. Although no donor site morbidities were observed in this case series, surgeons should be careful during harvesting and monitor patients closely for probable morbidities.

There is controversy regarding whether distal clavicle excision (known as the Mumford procedure) should be a routine part of AC joint separation surgery. Beaver *et al* developed a biomechanical model to explore distal clavicle excision and determined that such excision in ACD was not associated with either anteroposterior or superior-inferior translation^25^. However, Dawson *et al* raised concerns about the possibility of CC ligament reconstruction failure when distal clavicle excision was added to the surgery and advised against excision to increase anteroposterior stability^26^. Although degenerative AC joint arthritis was evident in two patients (sixth and eighth patients), distal clavicular excision was not performed, which resulted in mild residual pain during heavy activities. Drawing on the experience gained from these patients, distal clavicle excision (the Mumford procedure) was utilised with the patients with pre-operative AC joint arthritis, as seen in the sixteenth and seventeenth cases. As a result, the UCLA rating scale scores improved to good/excellent from fair/poor in this group of patients. Due to the conflicting results reported in the literature, we recommend that distal clavicle excision should not be performed routinely. However, if AC joint arthritis is present, distal clavicle excision should be considered. Distal clavicle excision surgery is a widely accepted intervention for AC joint arthritis in the elderly, and it can be a good choice to help prevent patient dissatisfaction and chronic pain^27,28^.

This study is not without limitations. First, the sample size was small, and studies with larger samples are needed to further validate the technique. Due to the retrospective design of the study, there were difficulties related to data collection. Although successful results were obtained using the technique employed in this study, a control group was lacking to allow comparisons, which is why we compared our results with those reported in the literature.

## Conclusion

The current study demonstrates that this technique is simple, reliable, biologic and dynamic surgical procedure with no major complications. It is also a cost-effective procedure, as it can be performed with K-wires and autogenous STGs. It has a major advantage of leaving no implants inside the joint, which could result in complications, and it can be performed in basic operating room settings. Further, it may be combined with distal clavicle resection in elderly patients with AC joint arthritis.
